# A systematic review of the potential effects of medications and drugs of abuse on dopamine transporter imaging using [^123^I]I-FP-CIT SPECT in routine practice

**DOI:** 10.1007/s00259-023-06171-x

**Published:** 2023-02-27

**Authors:** Youssef Chahid, Zulfiqar H. Sheikh, Max Mitropoulos, Jan Booij

**Affiliations:** 1 Amsterdam UMC location University of Amsterdam, Radiology and Nuclear Medicine, Meibergdreef 9, Amsterdam, The Netherlands; 2 Amsterdam UMC location University of Amsterdam, Clinical Pharmacy, Meibergdreef 9, Amsterdam, The Netherlands; 3 GE Healthcare, Pharmaceutical Diagnostics, Nightingales Ln, Chalfont Saint Giles, United Kingdom

**Keywords:** [^123^I]I-FP-CIT, DaTSCAN, Dopamine transporter imaging, Drug interactions

## Abstract

**Purpose:**

In routine practice, dopamine transporter (DAT) imaging is frequently used as a diagnostic tool to support the diagnosis of Parkinson’s disease or dementia with Lewy bodies. In 2008, we published a review on which medications and drugs of abuse may influence striatal [^123^I]I-FP-CIT binding and consequently may influence the visual read of an [^123^I]I-FP-CIT SPECT scan. We made recommendations on which drugs should be withdrawn before performing DAT imaging in routine practice. Here, we provide an update of the original work based on published research since 2008.

**Methods:**

We performed a systematic review of literature without language restriction from January 2008 until November 2022 to evaluate the possible effects of medications and drugs of abuse, including the use of tobacco and alcohol, on striatal DAT binding in humans.

**Results:**

The systematic literature search identified 838 unique publications, of which 44 clinical studies were selected. Using this approach, we found additional evidence to support our original recommendations as well as some new findings on potential effect of other medications on striatal DAT binding. Consequently, we updated the list of medications and drugs of abuse that may influence the visual read of [^123^I]I-FP-CIT SPECT scans in routine clinical practice.

**Conclusion:**

We expect that a timely withdrawal of these medications and drugs of abuse before DAT imaging may reduce the incidence of false-positive reporting. Nevertheless, the decision to withdraw any medication must be made by the specialist in charge of the patient’s care and considering the pros and cons of doing so.

**Supplementary Information:**

The online version contains supplementary material available at 10.1007/s00259-023-06171-x.

## Introduction

Dopamine transporter (DAT) imaging is a diagnostic tool to support the diagnosis of Parkinson’s disease (PD) by determining if there is loss of functional nigrostriatal dopaminergic neurons [1]. The application helps to differentiate between parkinsonian syndromes characterised by dopaminergic degeneration, such as PD, from movement disorders not characterised by dopaminergic degeneration, such as essential tremor [2]. In routine practice, [^123^I]I-FP-CIT (or [^123^I]ioflupane; marketed as DaTSCAN) is frequently used for DAT single-photon emission computed tomography (SPECT) imaging. This radiopharmaceutical is also used to differentiate dementia with Lewy bodies (DLB) from Alzheimer’s disease and is one of the recommended biomarkers to aid the diagnosis of DLB in the current 4^th^ consensus criteria for the diagnosis of DLB [3]. The role of DAT imaging in neurodegenerative disorders, such as PD and DLB, in clinical practice and research has been described in detail in a recent review by Wallert et al. [4].

In 2008, we published on the possible effects of medication on the visual interpretation of DAT imaging in routine clinical practice [5]. This publication has been well received, as this review has been cited more than 100 times (118; Web of Science citations in November 2022), and the information is also regularly used in guidelines to address the important clinical question of which medications or drugs of abuse should be considered for withdrawal before acquiring DAT imaging in routine practice to prevent misinterpretation due to the use of these medications [6–10]. Because this original publication was published almost 15 years ago [5], we were interested in reviewing the literature published since 2008 to evaluate whether additional information on this topic has become available.

## Methods and materials

We performed a systematic review of literature to examine which medications or drugs of abuse may influence striatal DAT imaging using SPECT or positron emission tomography (PET) tracers. We used the Preferred Reporting Items for Systematic Reviews and Meta-analyses (PRISMA) guideline for literature selection [11].

### Literature search strategy

**Fig. 1:**
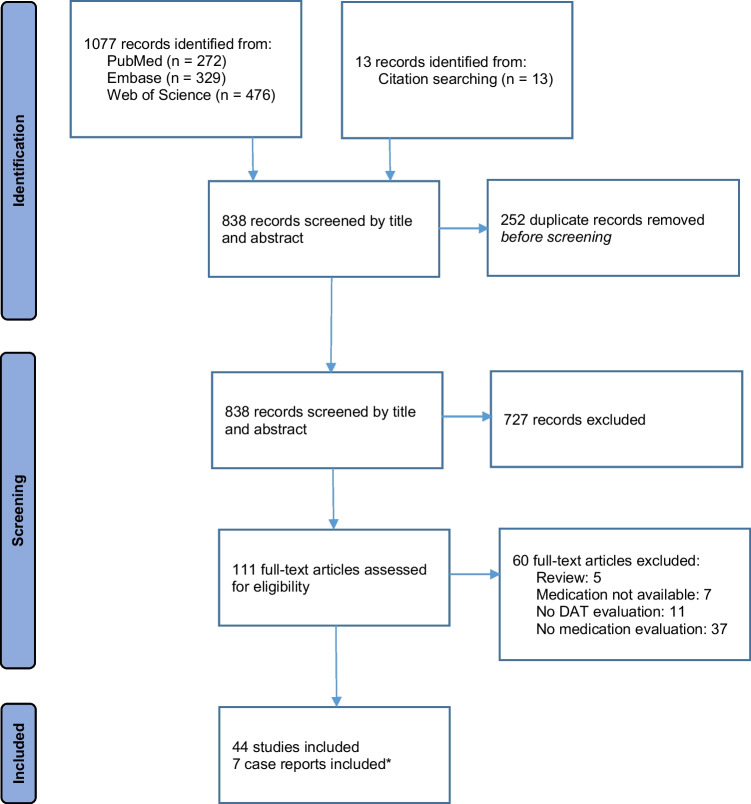
PRISMA flow diagram of included studies. * Case reports were only used to illustrate our recommendations to withdraw medications

To select relevant literature, we performed a systematic search for publications in PubMed, Embase, and Web of Science databases from January 2008 to November 2022. The selected terms used for the search are listed in the supplemental material. There were no limitations on language. A total of 838 potential studies were identified based on the search strategy (Fig. 1).

### Inclusion and exclusion criteria

All studies which examined medications and drugs of abuse, including the use of nicotine or alcohol, that may potentially influence striatal DAT imaging using SPECT or PET tracers were selected. Conference abstracts and studies not conducted in humans were excluded. Case reports which appeared in the search were only used to illustrate our recommendations to withdraw particular medications. We excluded medications that were not approved by the European Medicines Agency (EMA) or Food and Drug Administration (FDA). We also excluded chemicals/toxins that may influence striatal [^123^I]I-FP-CIT binding, e.g., carbon monoxide intoxication, industrial toxins, and pesticides, since such compounds are not generally encountered in routine clinical practice and frequently cannot be easily withdrawn. However, abuse substances (such as amphetamines, smoking and alcohol) were included, as physicians often encounter this with their patients, and patients could be advised to stop these before DAT imaging scan, where needed.

### Literature selection

Publications from the systematic search were imported on the Rayyan collaborative review platform [12]. After removal of duplicates, the title and abstract were screened by two reviewers (YC and MM), and eligible studies were selected for full-text review. In the full-text review, studies that did not measure the effect of medications or drugs of abuse on the striatal binding of DAT radiotracers were removed. Finally, we carefully examined the reference list of the selected full-text publications to identify additional studies that met the inclusion criteria.

### Outcome measure

The main aim of this review is to evaluate whether there is new or additional evidence in the literature regarding whether medications or drugs of abuse, including the use of nicotine and alcohol, can influence the visual read of [^123^I]I-FP-CIT SPECT scans performed in routine practice. In accordance with the definition used in our original review on this topic in 2008, it may be reasonable to assume that at least 20% occupancy of striatal DAT by medications or drugs of abuse may be required to cause a misleading interpretation of the visual assessment of [^123^I]I-FP-CIT SPECT scans [5].

## Results

### Anti-Parkinson medication

**Fig. 2:**
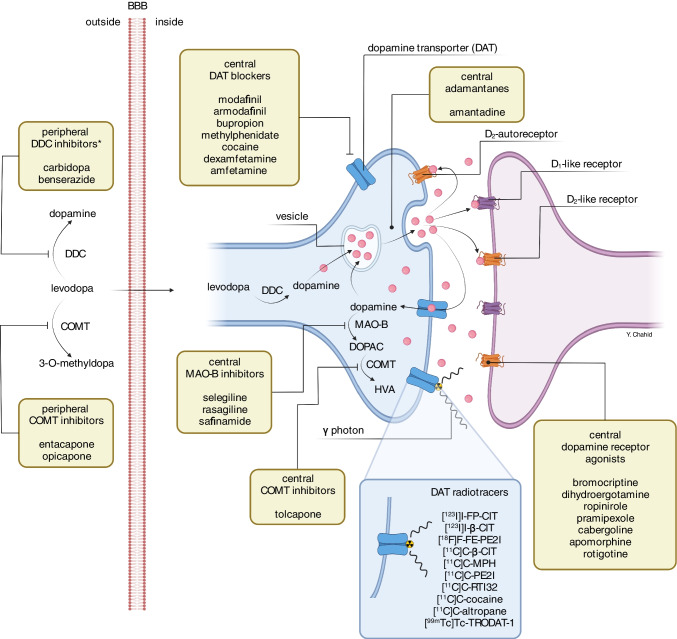
Illustration of a terminal dopaminergic neuron as well as a postsynaptic neuron at the level of the striatum (right panel). This illustrates where dopamine transporters and receptors are predominantly expressed and the mechanism of action of commonly used anti-Parkinson medication. It also provides a list of typical dopamine transporter blockers and radiotracers that bind to the dopamine transporter. BBB: blood‒brain barrier; DAT: dopamine transporter; DDC: DOPA decarboxylase; COMT: catechol-O-methyltransferase; MAO-B: monoamine oxidase B; DOPAC: 3,4-dihydroxyphenylacetic acid; HVA: homovanillic acid

In Fig. 2, the main mechanism of action of different classes of anti-Parkinson medication is described. In the treatment of PD, levodopa (a precursor of dopamine) is used to increase dopamine concentrations in the central nervous system [13]. Levodopa crosses the protective blood‒brain barrier (BBB), whereas dopamine itself cannot. The therapeutic benefit of using peripheral dopa-decarboxylase (DDC) inhibitors and catechol-O-methyltransferase (COMT) inhibitors is based on preventing the peripheral metabolism of levodopa to dopamine and 3-O-methyldopa, respectively, thus increasing the bioavailability of levodopa for crossing the BBB. While dopamine agonists directly bind preferentially to dopamine D_2_-like receptors, levodopa is converted in the central nervous system by the enzyme DDC to dopamine before it is temporarily stored within dopaminergic neuron vesicles and released in the synaptic cleft, where it interacts with dopamine receptors. Central acting COMT inhibitors and monoamine oxidase B (MAO-B) inhibitors prevent the metabolism of dopamine (Fig. 2).

In our 2008 review, we described that we did not recommend withdrawing dopaminergic medication, such as levodopa, dopamine agonists, COMT inhibitors, and MAO-B inhibitors, prior to DAT imaging in routine practice [5]. This recommendation is still valid and supported by the results of several clinical studies [14–25]. Our present search of the literature did not find new evidence that typical dopaminergic anti-Parkinson medication can reduce the visual assessment of DAT. Therefore, we still do not recommend withdrawing this class of medications in the preparation phase of DAT imaging in routine practice (Table 1). This does not, however, exclude that such medications may induce small effects on quantification, which may be relevant for scientific studies [25].

**Table 1:** Anti-Parkinson medications with no evidence of significant effect on DAT imaging

Medication*
Dopamine agonists bromocriptine dihydroergotamine ropinirole pramipexole cabergoline apomorphine rotigotine
MAO-B inhibitors selegiline rasagiline safinamide
DDC inhibitors carbidopa benserazide
COMT inhibitors entacapone opicapone tolcapone
Adamantanes amantadine memantine
Anticholinergic agents trihexyphenidyl biperiden orphenadrine benztropine

* Based on published study data on drug interference between January 2008 and November 2022

Amantadine and memantine are frequently used to treat dyskinesia in PD and cognitive impairments in PD or DLB, respectively [26, 27]. We could not find clinical DAT imaging studies that assessed the effects of these drugs on DAT binding.

Anticholinergic drugs, such as benztropine or trihexyphenidyl, are frequently used, particularly in young parkinsonian patients, and cholinesterase inhibitors are frequently used in demented parkinsonian patients, including DLB patients [28]. While benztropine has a modest affinity for DAT, the affinity of trihexyphenidyl is in the low micromolar range [29]. We described earlier that benztropine significantly occupied the striatal DAT in monkeys [29]. However, after 2008, no DAT imaging study showed that benztropine could indeed significantly influence in vivo striatal DAT binding, and no case report on false-positive findings in patients was published. Additionally, other anticholinergics, such as scopolamine, orphenadrine, or trihexyphenidyl, may induce *increased* [^123^I]I-FP-CIT binding to striatal DATs, which may influence quantification but not the visual assessment of DAT scans [5]. Overall, there is no evidence that benztropine will significantly decrease [^123^I]I-FP-CIT binding in humans; therefore, we now list this drug in Table 1.

### Stimulants such as cocaine, amphetamines, and adrenergic agents

In our original paper, we explained why it is likely that the use of DAT blockers such as cocaine and amphetamines will influence the visual analysis of [^123^I]I-FP-CIT SPECT scans [5]. It is well known that cocaine blocks striatal DAT significantly in the living human brain (60–77%) [30]. Additionally, abusers of dexamphetamine and methamphetamine show a significant reduction in striatal DAT binding compared to healthy controls [31–36]. Of note, although cocaine is a classic DAT blocker, amphetamines are much weaker DAT blockers, but this kind of drug may also induce a fast internalization of the DAT, which results in lower striatal DAT binding [37, 38]. To illustrate the potential effects of amphetamines on the visual analysis of [^123^I]I-FP-CIT SPECT scans, a recent case report suggested that the use of amphetamines can indeed induce false-positive findings (abnormal scan) on the visual read of an [^123^I]I-FP-CIT SPECT scan [39]. Therefore, we still recommend not performing DAT imaging in patients who are currently using cocaine or amphetamines (Table 2).

**Table 2:** Medications that significantly decrease the striatal binding  of [^123^I]I-FP-CIT

Medication	Stop prior DAT imaging *
Centrally acting drugs	
Cocaine Dexamphetamine Methamphetamine Phentermine Ephedrine Methylphenidate Dexmethylphenidate Modafinil Armodafinil	1 day 2 days 1 day 5 days 1 day 1 day 1 day 3 days 3 days
Opioids	
Fentanyl Codeine	2 day 1 day
Antipsychotics	
Haloperidol	5 days
Antidepressants	
Bupropion	5 days

* We assume that drug interactions are reversible and concentration dependent. Stop the medication at least 5 times the half-life prior to DAT imaging [40–52]. However, the normalisation of DAT binding may take longer in some cases, such as drug interactions (inhibiting drug metabolism) and some genotypes (poor metabolisers). See also the Discussion section on this topic

Medications such as phentermine or ephedrine are stimulants that are structurally closely related to amphetamines and are used to suppress appetite and consequently to lose weight. In our original paper, we explained why it is likely that the use of these amphetamine-like drugs may influence the visual analysis of [^123^I]I-FP-CIT SPECT scans [5]. In line with this rationale, a recent case report showed that the use of phentermine may indeed induce a false-positive [^123^I]I-FP-CIT SPECT scan [53]. Therefore, these kinds of drugs are listed in Table 2.

A major mechanism of action of methylphenidate and its diastereoisomer dexmethylphenidate is blockage of DAT (Fig. 2). Indeed, one hour after a single dose of methylphenidate, approximately 60% of striatal DAT binding is blocked, as measured in 12 healthy subjects with [^11^C]C-altropane PET [54]. Additionally, Spencer et al. reported similar results of approximately 40% striatal DAT occupancy at 10 h post administration of 38 mg methylphenidate in 21 healthy volunteers [55]. The same research group found similar effects of 45% striatal DAT occupancy after a single dose of dexmethylphenidate in 18 healthy subjects [56]. The study by Vles et al. reported that 3 months after the initiation of methylphenidate treatment in 6 children, a reduction of 58% in striatal [^123^I]I-FP-CIT binding was observed [57]. Crunelle et al. showed that 30 mg methylphenidate reduced [^123^I]I-FP-CIT binding in the caudate nucleus with 44% in ADHD patients (*n* = 16) and 37% in ADHD patients (*n* = 8) with comorbid cocaine dependence [58]. This is in line with the 52% DAT reductions at the left and right caudate nucleus after 3 weeks of methylphenidate treatment in the [^99m^Tc]Tc-TRODAT-1 study of Szobot and colleagues [59]. Interestingly, more recent studies have also shown similar results. The use of methylphenidate was associated with a 30% decrease in striatal [^123^I]I-ß-CIT binding in 13 ADHD patients [60]. Only 2.5 h after oral intake of 30 mg methylphenidate, the striatal binding of [^123^I]I-FP-CIT was reduced by 43% in 13 patients with traumatic brain injury [61]. Akay and co-workers found that 2 months of methylphenidate (1 mg/kg/day) treatment in 20 adolescents with ADHD resulted in 7% and 9% reductions in [^99m^Tc]Tc-TRODAT-1 binding in the right caudate nucleus and right putamen, respectively [62]. Two different case reports were published that showed false-positive results on visual reads of [^123^I]I-FP-CIT scans, likely due to methylphenidate use [53, 63]. Therefore, methylphenidate and dexmethylphenidate are also listed in Table 2.

Administration of modafinil and its R-enantiomer, armodafinil, will influence the visual assessment of DAT imaging. The binding of modafinil to DAT was assessed with positron emission tomography (PET) in 27 cocaine-dependent patients. The [^11^C]C-PE2I binding in the striatum was reduced with 82% in patients using 100 mg modafinil daily for 2 weeks, whereas placebo had no significant effect [64]. Another [^11^C]C-PE2I PET study showed a mean striatal DAT occupation of 51% and 57% in 10 healthy volunteers after administration of 200 mg and 300 mg modafinil, respectively [65]. These findings are in line with the results of the research group of Volkow and colleagues [66]. They reported 2 h after 200 mg or 400 mg modafinil administration a decrease in [^11^C]C-cocaine binding in the caudate (54%), putamen (47%), and nucleus accumbens (39%) in 10 healthy volunteers [66]. Additionally, a case report described that the use of modafinil resulted in a false-positive [^123^I]I-FP-CIT SPECT scan [67]. Spencer et al. showed, in a study with 12 subjects, that administration of armodafinil reduced striatal DAT binding with 65% as assessed with [^11^C]C-altropane PET [68]. Therefore, we have added both modafinil and armodafinil to the list of medications that we advise to consider withdrawal before performing DAT imaging in routine practice (Table 2).

In 2008, we recommended, based on preclinical data, withdrawing adrenergic agonists such as phenylephrine and norepinephrine [5]. However, we found only one study that examined the DAT occupancy of dl-methylephedrine, a derivative of ephedrine, in healthy controls [69]. They showed that the striatal DAT occupancy was only 4.4 and 3.6% for the caudate nucleus and putamen, respectively. Therefore, we do not recommend withdrawing adrenergic agonists when performing DAT imaging.

### Opioids

In our original paper, we explained why we believe that fentanyl may influence the interpretation of DAT imaging [5]. Bergstöm et al. reported that [^123^I]I-β-CIT binding in the basal ganglia of a female patient was decreased by 37% after fentanyl administration compared to binding after a 2-week drug-free period [70]. Interestingly, in the study by Hou et al., 22 subjects who were addicted to codeine-containing cough syrup were studied and compared to 27 healthy age-matched controls [71]. They showed that the striatal [^99m^Tc]Tc-TRODAT-1 SPECT binding ratios were 35% lower in the codeine group. Based on these data, we listed fentanyl and codeine in Table 2.

In heroin addicts, striatal DAT binding may be significantly lower than in matched controls [72–74]. However, in these kinds of studies, the subjects are typically abstinent for heroin for at least a short period. This may indicate that the disorder itself may induce lower DAT binding, or alternatively, the lower striatal DAT binding may play a role in the aetiology of the disorder (see also the Discussion on this topic). Nevertheless, we could not find literature on studies performed in humans that indicates that the acute use of heroin may induce lower DAT binding.

### Antipsychotics

All antipsychotics are dopamine receptor antagonists, and antipsychotics, such as quetiapine, are associated with a relatively low risk for inducing parkinsonism and are frequently prescribed to psychotic PD patients [75]. In 2008, we did not recommend withdrawing antipsychotics when performing DAT imaging [5]. This recommendation was based on the studies of Lavalaye et al. and Mateos and co-workers [76, 77]. They reported that olanzapine and risperidone did not influence the striatal DAT binding of [^123^I]I-FP-CIT [76, 77]. A more recent [^99m^Tc]Tc-TRODAT-1 SPECT study by Chang et al. confirmed that no significant difference was noticed in the striatal DAT occupancy between baseline and 6 months of antipsychotic treatment [78]. However, Schmitt et al. examined striatal DAT binding using [^99m^Tc]Tc-TRODAT-1 SPECT in first-episode schizophrenic patients who were treated for 2 weeks with haloperidol and compared this group with neuroleptic-naïve schizophrenic patients as well as a group of healthy controls [79]. Remarkably, the 12 patients on haloperidol had 25% lower striatal DAT binding than the 12 healthy controls and 12 neuroleptic-naïve patients. Consequently, we have now only added haloperidol to Table 2. However, we could not find evidence that other antipsychotics significantly reduce DAT binding in humans.

### Antidepressants

In 2008, we recommended withdrawing the antidepressant bupropion when a patient is referred for DAT imaging [5]. Bupropion is frequently prescribed as an antidepressant or as an antismoking drug. Kugaya et al. performed a [^123^I]I-β-CIT study with 8 healthy controls and found no significant effects of bupropion on striatal DAT binding [80]. In contrast, Argyelán et al. showed with a [^99m^Tc]Tc-TRODAT-1 SPECT study that after 4 weeks of bupropion treatment, a 21% decrease in striatal DAT binding ratios was induced in 9 depressed patients [81]. These findings were confirmed in another [^99m^Tc]Tc-TRODAT-1 SPECT study performed in 23 patients with major depression [82]. Additionally, a PET study with [^11^C]C-RTI32 in 8 depressed patients reported that the striatal DAT occupancy after bupropion treatment was 14% decreased [83]. Another PET study with [^11^C]C-β-CIT-FE performed in 6 healthy controls showed that 24 h after the last dose of 150 mg bupropion SR, the average DAT occupancy was 25% [84]. Indeed, our recommendation to withdraw bupropion prior to DAT imaging is substantiated by reports of false-positive interpretations of [^123^I]I-FP-CIT SPECT scans of patients on this drug [85–87]. Therefore, bupropion is listed in Table 2.

As addressed earlier, [^123^I]I-FP-CIT binds with high affinity to DAT but also shows a modest affinity for the serotonin transporter (SERT) [88]. Indeed, several clinical studies evaluated the effects of the use of selective serotonin reuptake inhibitors (SSRIs) on DAT imaging. The striatal DAT availability did not significantly change in 8 patients with major depressive disorder with [^99m^Tc]Tc-TRODAT-1 after 24 weeks of antidepressant treatment (paroxetine, sertraline, venlafaxine and fluoxetine) [89]. We also experimentally studied the influence of paroxetine on [^123^I]I-FP-CIT binding to DATs in the striatum in a double-blind, placebo-controlled, crossover study with 8 healthy young male controls. Compared with placebo, 20 mg paroxetine (taken orally 2 and 27 h before DAT imaging) increased the striatal-to-nonspecific [^123^I]I-FP-CIT binding ratios by 9% [88]. Similar small increases in striatal DAT binding were also reported in the [^11^C]C-PE2I PET study of Hjorth and co-workers. In that study, 27 patients were treated with 20 mg escitalopram per day for a period of 9 weeks [90]. Makkonen et al. examined the effects of 6 months treatment with fluoxetine 10–40 mg daily on striatal DAT binding, as indexed with [^123^I]I-ß-CIT SPECT (Fig. 2), in 13 children suffering from autism [91]. The authors described no change or a slight increase in striatal binding in the subgroup of 7 children who did not respond clinically and a small decrease in the 6 children who did improve clinically [91]. Ziebell et al. showed no significant effect of *acute* 0.15 mg/kg citalopram infusion on [^123^I]I-PE2I binding in the striatum [92]. The same treatment, however, resulted in a 23% reduction in [^123^I]I-FP-CIT binding in the striatum [92]. On the other hand, Rominger et al. showed that the striatal DAT binding of [^123^I]I-ß-CIT SPECT was, compared to baseline measurement, 20% increased after 6 weeks of treatment with approximately 15 mg escitalopram per day in a group of 19 depressed patients [93]. This effect was significant in both the caudate nucleus and putamen. Warwick et al. also examined the effects of escitalopram on striatal [^123^I]I-FP-CIT binding in patients suffering from social anxiety disorder [94]. After 12 weeks of treatment with 20 mg escitalopram daily, the [^123^I]I-FP-CIT binding increased significantly only in the left striatum. However, the results of the double-blind trial with escitalopram of Zoons et al. showed that a 6-week treatment course with 10 mg escitalopram daily did not influence the DAT binding of [^123^I]I-FP-CIT in 8 patients with cervical dystonia [95]. Krause and colleagues did not show a significant influence of 6 weeks treatment with escitalopram on striatal [^123^I]I-β-CIT binding in a group of 19 patients with major depression [96]. Since (sub)chronic treatment with escitalopram induces no significant decrease in striatal DAT binding and possible increases in binding are observed both in the putamen and caudate nucleus, it is unlikely that the use of this SSRI will negatively influence a visual read of DAT imaging. Finally, Seo et al. showed that the striatal-to-occipital cortex binding ratio, measured with [^18^F]F-FP-CIT PET, was not significantly different between SSRI users (escitalopram (*n* = 19) or fluvoxamine (*n* = 20)) and non-SSRI users (clonazepam (*n* = 72)) [97]. Therefore, based on the conflicting clinical studies mentioned above, we do not recommend withdrawing SSRIs before DAT imaging in routine clinical practice.

### Other medications

The study by Ikeda et al. showed no influence of 50 mg/day of the antiepileptic drug zonisamide on striatal DAT binding in a [^123^I]I-FP-CIT SPECT study in 15 PD patients [98]. Monti and colleagues studied the effect of N-acetyl cysteine (50 mg/kg/week intravenous or 1200 mg/day oral) on striatal [^123^I]I-FP-CIT binding in 12 patients with PD. This clinical study showed significantly increased DAT binding in the caudate and putamen of 4% and 8%, respectively [99]. The same research group repeated the study in 24 patients with PD several years later. They again reported small but significantly increased [^123^I]I-FP-CIT binding in the caudate nucleus and putamen of 3% and 8%, respectively [100]. Although both observations on DAT binding were statistically significant, a small increase in striatal [^123^I]I-FP-CIT binding will not influence the visual read of [^123^I]I-FP-CIT SPECT scans in routine practice, and consequently, we do not recommend withdrawing N-acetyl cysteine before DAT imaging in routine practice.

### Smoking and alcohol

The potential effect of smoking or nicotine therapy on striatal DAT binding is unclear. An observational study by Itti and co-workers showed a slower decrease in striatal [^123^I]I-FP-CIT binding in 6 PD patients on nicotine therapy (-4%) than expected in PD patients (-10% per year) [101]. However, Gigante et al. found a significantly lower [^123^I]I-FP-CIT binding in the putamen of 11% in 13 current smokers compared to non-smokers [102]. Interestingly, the largest study by Thomsen and colleagues on this topic compared 64 non-smokers, 39 ex-smokers and 26 current smokers and found no statistically significant difference in striatal [^123^I]I-FP-CIT binding between the different groups [103]. Consequently, there is no clear evidence that smoking will influence the visual read of [^123^I]I-FP-CIT SPECT scans in routine practice.

It is also not clear whether alcohol use affects the visual analysis of [^123^I]I-FP-CIT SPECT scans. Cosgrove and co-workers showed that the mean striatal [^123^I]I-β-CIT binding was 16% higher in 14 heavy alcohol drinkers than in 14 controls [104]. In that study, heavy drinkers were scanned during acute withdrawal (between 1 and 5 days after their last drink). These findings are in line with the observation of Addolorato and co-workers in an [^123^I]I-FP-CIT SPECT study with 14 alcohol-dependent patients (scanned 24 h after their last drink) and 20 healthy controls. They reported significantly increased [^123^I]I-FP-CIT binding in the caudate nucleus and putamen of 15% and 13%, respectively [105]. In contrast, another study examined 26 alcohol-dependent patients (last drink less than 48 h before DAT imaging) and 22 healthy volunteers and showed a 20% decrease in striatal [^99m^Tc]Tc-TRODAT-1 binding [106]. Additionally, Grover et al. found a decrease of 26% in striatal [^99m^Tc]Tc-TRODAT-1 binding in 20 alcohol-dependent patients (last drink consumed within 30 days preceding the DAT scan) compared to 20 healthy volunteers [107]. Based on the conflicting clinical studies of the effect of alcohol on striatal DAT binding, we do not recommend withdrawing the use of alcohol before DAT imaging in routine clinical practice.

## Discussion

Here, we updated which specific medications and drugs of abuse might significantly influence the visual read of [^123^I]I-FP-CIT SPECT scans as performed in routine practice. Compared to our original review, we found new evidence that the use of some medications, such as haloperidol and codeine, might influence such a read. Additionally, we found additional support that medications that we initially recommended to withdraw before performing such a visual read could indeed induce a false-positive scan (e.g., bupropion).

In addition to visual interpretation, semi-quantitative analysis is recommended in EANM/SNMMI guidelines to objectively assess striatal DAT binding [6, 7]. Although semi-quantification is very useful to combine with the visual read as a tool to provide a more objective analysis of DAT scans, we believe that a careful visual evaluation of DAT scans should always be done (even when using quantification), and in some hospitals, it is the only method used [108–111]. Therefore, we focused on drugs that may influence significantly the visual read of [^123^I]I-FP-CIT SPECT imaging (and the semi-quantification as well).

We did not find new evidence that anti-Parkinson medication may influence the visual assessment of DAT imaging. However, this does not mean that commonly used dopaminergic medication in PD cannot influence DAT imaging quantitatively. For example, Rossi et al. showed that 12 weeks of treatment with the dopamine receptor agonist rotigotine (mean dose of 7.75 mg/day) in early PD patients may increase [^123^I]I-FP-CIT binding by 13 and 17% in the caudate nucleus and putamen, respectively [111]. Although of scientific interest, this small increase will not significantly influence the visual reporting of DAT imaging in routine practice since this visual analysis is primarily based on the detection of differences in putamen versus caudate nucleus binding as well as asymmetry [112, 113]. However, this small effect on striatal [^123^I]I-FP-CIT binding may influence the semi-quantitative analysis in routine practice using software such as DaTQUANT or BRASS [114, 115]. However, it is likely that such potential small effects on quantification will influence both the binding in the caudate nucleus and putamen to a similar extent but not the asymmetry of binding. Therefore, when a scan is judged as normal on the visual read but the quantitative analyses are borderline normal for uptake in both the caudate nucleus and putamen, it might be helpful to carefully check which drugs were used by the patient during scanning.

In this review, we focused primarily on the findings of medication on DAT imaging studies performed in humans. We did so because we believe that these findings are more relevant for DAT imaging studies performed in routine clinical practice than findings from animal studies. A striking example is studies on the effects of levodopa. In this regard, on the one hand, Nikolaus and colleagues showed in 2 different studies, performed in rats, that 5 mg/kg levodopa decreased striatal [^123^I]I-FP-CIT binding by 24–34% [116, 117]. On the other hand, the Parkinson Study Group found a much smaller effect of levodopa in patients with PD [25]. They studied the effects of levodopa/carbidopa on the progression of PD. The striatal [^123^I]I-β-CIT binding was only 2.6–5.8% decreased among 90 PD patients who received levodopa (150–600 mg/day) compared with 26 PD patients receiving placebo [25]. Additionally, Schillaci et al. studied 15 PD patients under stable levodopa/carbidopa therapy and after at least 20 days of treatment wash-out, and they did not find a significant difference in striatal [^123^I]I-FP-CIT binding ratios [17]. Overall, these findings illustrate that it is unlikely that the use of levodopa will interfere significantly with the visual read of DAT imaging but that (small) quantitative effects may exist. It also illustrates that findings in control rats cannot be simply generalised to DAT imaging studies in parkinsonian patients.

Schmitt et al. showed that schizophrenic patients on haloperidol had 25% lower striatal [^99m^Tc]Tc-TRODAT-1 binding than controls and neuroleptic-naïve patients [79]. It should be noted that the haloperidol-treated patients were not scanned before starting treatment in this study. Although understandable since it is hard to scan schizophrenic patients in the neuroleptic-naïve state, one cannot exclude the possibility that this subgroup of patients already had lower striatal DAT expression than the neuroleptic-naïve group at baseline (i.e., before they started haloperidol treatment). Nevertheless, the decision to withdraw haloperidol must always be made by the specialist in charge of the patient’s care and taking into account the pros and cons of doing so.

Interestingly, Hou et al. showed that the striatal [^99m^Tc]Tc-TRODAT-1 SPECT binding ratios were 35% lower in subjects who were addicted to codeine-containing cough syrup compared to control data obtained in healthy controls [71]. Unfortunately, it is not clear whether these patients were abstinent when they were scanned. This is relevant since lower striatal DAT binding is frequently described in all kinds of substance use disorders, even when they are abstinent. For example, striatal [^123^I]I-FP-CIT or [^11^C]C-cocaine binding is lower in abstinent heroin- and methamphetamine-dependent users [72, 118]. Additionally, apart from codeine-containing cough syrup, codeine-containing tablets are frequently used as pain medication. In this regard, the prevalence of pain is high in PD, and codeine is frequently prescribed in the treatment of pain in PD patients [119]. Therefore, it might be of interest to examine whether administration of codeine indeed may influence striatal [^123^I]I-FP-CIT binding in PD by performing a double-blind, placebo-controlled study.

As mentioned earlier, striatal DAT binding may be lower in abstinent subjects suffering from substance use disorders [72, 118]. In this regard, it is interesting that we found 4 clinical studies that evaluated the effects of alcohol consumption on striatal DAT binding in heavy drinkers [104–107]. At least 3 of these studies were performed during acute alcohol withdrawal, and they showed mixed results on striatal DAT binding, with 2 studies showing increased striatal DAT binding [104, 105]. Interestingly, one study published in 1999 (and consequently not included in our selection of studies) examined both the effects of acute alcohol withdrawal and a 4-week period of abstinence on striatal DAT binding in the same subjects [120]. They showed decreased striatal [^123^I]I-β-CIT binding during acute withdrawal but increased DAT binding (compared to control data) after a 4-week period of abstinence [120]. This may indicate that the effects on DAT in alcohol addiction may differ from findings in substance use disorders such as heroin or amphetamine addiction [72, 118]. Additionally, due to the mixed results reported on DAT binding, particularly during acute withdrawal, we do not recommend withdrawing alcohol intake when performing an [^123^I]I-FP-CIT SPECT scan in routine clinical practice.

Although we do not recommend withdrawing SSRIs when performing DAT imaging in clinical practice, it is likely that SSRIs may influence striatal [^123^I]I-FP-CIT binding ratios quantitatively, at least to a small extent, due to [^123^I]I-FP-CIT binding to the serotonin transporter outside of the striatum [88]. Additionally, a recent case report suggested that the use of venlafaxine may induce a false-positive [^123^I]I-FP-CIT scan in a DLB patient [119]. However, Shang et al. showed in an experimental study that 5 days of treatment with venlafaxine in 5 healthy controls induced a small *increase* in striatal [^123^I]I-β-CIT binding [121]. Taking into account the findings by Shang et al. and that most DAT imaging in humans did not show a significant effect on striatal DAT binding, we are not convinced that venlafaxine should be stopped prior to DAT imaging.

Some medications, that are mentioned in the SmPC of [^123^I]I-FP-CIT, are not included in this review because we could not find evidence from clinical trials to include them, such as amoxapine, buspirone, and norephedrine. Medications such as mazindol and phenylpropanolamine are not included since they are no longer marketed in Europe or USA. Also, in the literature, DAT imaging studies in humans have shown a significant blockage of DAT binding of newly developed drugs. For example, the potential antidepressant BMS-820836 or the anti-obesity drug tesofensine significantly block striatal DAT binding in vivo [122, 123]. However, we did not list these drugs in Table 2 because they are also not available on the market.

When interacting medication is discontinued, the normalisation of striatal DAT expression itself may occur gradually. We assume that *acute* drug interactions with DAT are reversible and concentration dependent. To wait at least 5 times the half-life of the interacting medication will be enough to perform DAT imaging since the medication will no longer occupy the DAT [9]. However, the metabolism of the interacting medication may take longer in some cases, such as due to other drug interactions (inhibition of metabolism of the interacting medication) and some genotype (poor metabolisers of the interacting medication) [124]. This can also be the case when interacting medications are used *chronically* and where other physiological feedback mechanisms have influenced the striatal DAT density. The time course for DAT normalisation to normal expression may be delayed compared to the time required for elimination of the interacting medication. In the case of medication-induced DAT *upregulation*, it should be noted that it may require weeks to return to baseline DAT density. The normalisation of the DAT density is likely to depend on the metabolism of the inducing medication and, more importantly, the natural degradation time of the DAT enzymes. For example, the half-life of rifampicin is only 2 to 5 h, but it has been reported that the normalisation time for midazolam clearance is 4 weeks after withdrawal of rifampicin [125]. Additionally, we do not recommend withdrawing medication, e.g., orphenadrine and trihexyphenidyl, that may increase the DAT binding of radiotracers such as [^123^I]I-FP-CIT simply because it is unlikely that a small systemic increase in binding (i.e., symmetrically in both the caudate nucleus and putamen) will negatively influence the visual interpretation of an [^123^I]I-FP-CIT SPECT scan.

A clear strength of this study is its systematic approach. Limitations are that few studies have been designed to evaluate which medication may influence in vivo DAT binding in PD or DLB patients. Examples of such studies are DAT imaging studies in early PD patients who evaluated the effects of (sub)chronic treatment with levodopa or dopamine agonists on striatal DAT binding in an experimental setting [17, 20, 111]. Such studies are very informative to address whether a drug should be withdrawn before performing DAT imaging. Ideally, all drugs that are frequently used in PD or DLB patients should be tested this way, but unfortunately, this is not a realistic approach considering the high costs and efforts to perform such studies. Consequently, in this systematic review, we frequently had to rely on the outcomes of cross-sectional studies or on studies in which the drug was only tested acutely, but not (sub)chronically, which limits the interpretation of whether or not this drug will influence the visual read of an individual DAT scan. Finally, we cannot exclude the possibility that the striatal DAT binding ratios with radiotracers such as [^11^C]C-PE2I and [^99m^Tc]Tc-TRODAT-1 are more or less sensitive to the effects of medication with [^123^I]I-FP-CIT binding ratios, e.g., due to differences in kinetics and/or image quality.

In this systematic review, we aimed to provide an overview of the current knowledge on the potential effects of medications, drugs of abuse, tobacco and use of alcohol on the visual read of DAT imaging in routine clinical practice. We hope that a timely withdrawal of these medications may reduce the incidence of false-positive findings on such scans. Nevertheless, the decision to withdraw any medication must always be made by the specialist in charge of the patient’s care and taking into account the pros and cons of doing so.

## Supplementary Information

Below is the link to the electronic supplementary material.

Supplementary file1 (DOCX 16 KB)

## Data Availability

The literature search and article selection are available from the corresponding author on reasonable request.

## Abbreviations

PD: Parkinson’s disease
DLB: Dementia with Lewy bodies
DAT: Dopamine transporter
PET: Positron emission tomography
SPECT: Single-photon emission computed tomography
