# Supplementary material for: A systematic review of the potential effects of medications and drugs of abuse on dopamine transporter imaging using [123I]I-FP-CIT SPECT in routine practice
Source: Eur J Nucl Med Mol Imaging. 2023 Feb 27;50(7):1974–87. doi: 10.1007/s00259-023-06171-x (PMC10199883; doi:10.1007/s00259-023-06171-x)
Supplement: Supplementary file 1 — Supplementary file1 (DOCX 16 KB) [file 259_2023_6171_MOESM1_ESM.docx]

**Supplement**

PubMed search terms:

("datscan*"[All Fields] OR "dat spect*"[All Fields] OR "dat pet*"[All Fields] OR ("dat"[All Fields] AND "radionuclide imaging"[All Fields]) OR ("FP-CIT"[All Fields] OR "b-cit"[All Fields] OR "beta-CIT"[All Fields] OR "PE2I"[All Fields] OR "trodat*"[All Fields] OR "RTI32"[All Fields] OR "c-cocaine"[All Fields] OR "c-MPH"[All Fields])) AND ("influence"[All Fields] OR "alter*"[All Fields] OR "interact*"[All Fields] OR "effect*"[All Fields] OR "change*"[All Fields] OR "false positive"[All Fields] OR "false negative"[All Fields]) AND ("medication*"[All Fields] OR "drug*"[All Fields] OR "cocaine"[All Fields] OR "tobacco"[All Fields] OR "alcohol*"[All Fields] OR "pharmacolog*"[All Fields])

PubMed results, filter human, period 01-01-2008 till 08-11-2022: 272 results

Embase search terms:

(‘datscan’/br OR ‘dat spect’/br OR ‘dat pet’/br OR (‘dat’/br AND ‘radionuclide imaging’/br) OR (‘FP-CIT’/br OR ‘b-cit’/br OR ‘beta-CIT’/br OR ‘PE2I’/br OR ‘trodat’/br OR ‘RTI32’/br OR ‘c-cocaine’/br OR ‘c-MPH’/br)) AND (‘influence’/br OR ‘alteration’/br OR ‘interaction’/br OR ‘effect’/br OR ‘change’/br OR ‘false positive’/br OR ‘false negative’/br) AND (‘medication’/br OR ‘drug’/br OR ‘cocaine’/br OR ‘tobacco’/br OR ‘alcohol’/br OR ‘pharmacology’/br) AND 'human'/de AND 'article'/it

Embase results, period 01-01-2008 till 08-11-2022: 329 results

Web of Science search terms:

((((((((((((ALL=(datscan*)) OR ALL=(dat spect*)) OR ALL=(dat pet*)) OR ALL=(FP-CIT)) OR ALL=(b-cit)) OR ALL=(beta-CIT)) OR ALL=(PE2I)) OR ALL=(trodat*)) OR ALL=(RTI32)) OR ALL=(c-cocaine)) OR ALL=(c-MPH) OR (ALL=(radionuclide imaging)) AND ALL=(dat))) AND (((((((ALL=(influence)) OR ALL=(alter*)) OR ALL=(interact*)) OR ALL=(effect*)) OR ALL=(change*)) OR ALL=(false positive)) OR ALL=(false negative)) AND ((((((ALL=(medication*)) OR ALL=(drug*)) OR ALL=(cocaine)) OR ALL=(tobacco)) OR ALL=(alcohol*)) OR ALL=(pharmacolog*))

Web of Science results, period 01-01-2008 till 08-11-2022: 476 results
